# Exposure Response Supports Therapeutic Drug Monitoring for Dabigatran Etexilate in Patients with Atrial Fibrillation

**DOI:** 10.1055/s-0039-1693486

**Published:** 2019-07-18

**Authors:** Bryan H. Simpson, David M. Reith, Natalie J. Medlicott, Alesha J. Smith

**Affiliations:** 1 School of Pharmacy, University of Otago, Dunedin, New Zealand; 2 Dean’s Department, Dunedin Medical School, University of Otago, Dunedin, New Zealand

**Keywords:** dabigatran etexilate, therapeutic drug monitoring, hemorrhage, stroke, population pharmacokinetic

## Abstract

**Background** Dabigatran etexilate has become widely used for the prevention of stroke in patients with nonvalvular atrial fibrillation (NVAF). Currently, there is limited information in real-world patients relating to dabigatran etexilate exposure and response.

**Methods** This retrospective cohort study used administrative health data for NVAF patients dispensed dabigatran etexilate between July 1, 2011 and December 31, 2015. Outcomes of cerebrovascular accident (CVA), systemic embolism, and hemorrhage were extracted. Simulated pharmacokinetic parameters were obtained using a published population pharmacokinetic model of dabigatran etexilate. Area under the curve calculated for a 24-hour period at steady state (AUC _ss_ ), the exposure parameter, was derived using these simulations and the dosing data and the exposure–response relationship were investigated. The risk of adverse outcomes at AUC _ss_ quartiles was compared using Poisson regression and expressed using incidence rate ratios (95% confidence interval) adjusted for known potential confounders.

**Results** In total, 2,660 NVAF patients had been dispensed dabigatran etexilate. For these patients there was a decreased risk of hemorrhage (0.51, 0.32–0.79) when dabigatran AUC _ss_ was in the second quartile range of 1.70 to 1.96 mg h/L and thromboembolism/CVA (0.34, 0.16–0.76) when in the third quartile range of 1.97 to 2.26 mg h/L. An increased risk of hemorrhage (1.68, 1.18–2.38) was observed when AUC _ss_ was in the fourth quartile range of 2.27 to 12.76 mg h/L.

**Conclusion** An exposure–response relationship for dabigatran etexilate was described, where the most effective response was observed when AUC _ss_ was in the range of 1.70 to 2.26 mg h/L. Hence, it is feasible to develop guidance for optimal dosing to improve outcomes for patients with NVAF.

## Background

The direct oral anticoagulant (DOAC) dabigatran etexilate has become widely used since its approval for the prevention of stroke in patients with nonvalvular atrial fibrillation (NVAF), 1 but has been associated with several adverse outcomes. 2 3 Despite this there is paucity of information for the dose response for this important medication.

Dabigatran etexilate is normally administered as a twice-daily fixed-dose regimen with the dosage modified by age and/or creatinine clearance, use of concomitant drugs, and thromboembolic risk versus bleeding risk. 4 As with other DOAC medications, dabigatran etexilate exhibits a more predictable pharmacokinetic and pharmacodynamics profile when compared with vitamin K antagonists. 5

Although dabigatran etexilate has been promoted as not requiring routine coagulation monitoring, this has become controversial. 6 Certainly, there are specific clinical situations where assessment of the anticoagulation effect may be required, such as: for those who are bleeding, before and after administration of the dabigatran-specific antidote idarucizumab (Praxbind), evaluation of therapy failure in case of thrombosis, renal failure, before emergency surgery, before potential thrombolysis in ischemic stroke, at extremes of bodyweight, concomitant use of drugs known to affect pharmacokinetics of dabigatran etexilate, and in cases of suspected nonadherence. 7 Moreover, it has been reported that if therapeutic drug monitoring (TDM) was undertaken major bleeds could be reduced by 30 to 40% when compared with well-controlled warfarin. 6

Currently, the Sponsor indicates that an increased risk of bleeding can possibly be detected via elevated coagulation tests such as thrombin time (TT), ecarin clotting time (ECT), and activated partial thromboplastin time (aPTT). 8 However, there are limitations to the aPTT, such as the test having limited sensitivity making it unsuitable for precise quantification of the anticoagulant effect and the ECT test not being readily available or useful in the absence of standardization means that there is limited utility of these tests in clinical practice. 9 TT is a very useful test for detecting low levels of dabigatran etexilate in plasma. 10 However, TT becomes rapidly unclottable in the presence of low dabigatran etexilate concentrations, and therefore cannot be used for the overall expected drug concentration measurement. 11 In relation to treatment failure, it has been reported that the Sponsor believes that due to the low number of endpoint events for venous thromboembolism (VTE) patients and the availability of only pharmacokinetic data from clinical trials, only a limited exposure–response analysis could be undertaken for VTE prevention. 12 Therefore, there has been no guidance provided for monitoring patients for possible subtherapeutic treatment. 12

Despite it now being feasible to determine dabigatran plasma concentrations, 13 14 the thresholds are yet to be validated to ensure that clinical decisions based on the plasma concentrations represent the balance between avoiding bleeding and preventing thrombosis. 9 While it has been shown that there is an association between plasma concentrations and bleeding risk, the clear cut-offs for bleeding and thromboembolism/cerebrovascular accident (CVA) risk are not yet established. 9 With no reported reference ranges for dabigatran etexilate TDM, further studies would help clarify this issue.

The aim of the present study was to investigate the relationship between dabigatran etexilate exposure and adverse response in real-world patients.

## Methods

### Identification of Study Cohort

This was a retrospective cohort study using administrative health data from New Zealand. The databases accessed were the Best Practice Intelligence (BPI) database operated by Best Practice Advocacy Centre Clinical Solutions, New Zealand 15 and the New Zealand Ministry of Health Pharmaceutical Collection 16 (PC). The BPI database is a secure, internet-based, reporting tool that uses data downloaded from the enrolled general practice patient electronic health record (EHR) and covers approximately 20% of the New Zealand population. The PC contains prescription details about pharmaceutical dispensing claims for dabigatran etexilate along with other prescribed medicines as well as information on gender, date of birth, age, ethnicity, deprivation index score, frequency, and quantity dispensed for all of the New Zealand population. The study population included patients with a diagnosis of NVAF, aged 18 years or older, had at least one dispensing of dabigatran etexilate during the study period between July 1, 2011 (when dabigatran etexilate became available in New Zealand) and December 31, 2015, serum creatinine measurements within 60 days before or 30 days after their first dispensing of dabigatran etexilate, and bodyweight measurements within 1 year before or after their first dispensing of dabigatran etexilate. If a patient had a diagnosis of VTE or deep-vein thrombosis concurrently recorded with NVAF during the study period, they were excluded. The information from different datasets were linked using each patient's encrypted National Health Index number (NHI; a life-long unique identifier for all interactions with the New Zealand health system) to ensure patient anonymity. Ethical approval was obtained from the University of Otago, New Zealand Ethics Committee (reference: HD15/054).

### Patient Covariates

Dispensed medications, patient demographic, and covariate data were extracted from the PC and BPI databases for those who meet the inclusion criteria. Data related to medications that are reported to have an impact on area under the curve (AUC; verapamil, amiodarone, and proton pump inhibitors) 17 when taken concomitantly with dabigatran etexilate were extracted from the PC database for patients who had both medications dispensed within 90 days. As it has also been reported that patients with heart failure have an increase in AUC, 17 data related to whether a patient has a diagnosis of heart failure while being treated with dabigatran etexilate were extracted from the BPI database. Patients were stratified into age groupings of under 65 years, 65 to 74 years, 75 to 79 years, and over 80 years to align to both regulatory and the categories used by the Sponsor to guide dosing. 4 18 19

#### Estimation of Renal Function

If multiple serum creatinine or bodyweight measurements were recorded, the measurement closest to the initiation of dabigatran etexilate initiation was used. Weight measurements more than 5 standard deviations from the mean were considered to be due to data-entry errors and were excluded.

Baseline renal function was estimated via the Cockcroft–Gault equation using Eq. 1 :



where CrCl = creatinine clearance; age = age in years; weight = weight in kg; SCr = serum creatinine (expressed in mg/dL).

Eq. 1

#### Derived area under the curve at steady state (AUC _ss_ )

Simulated pharmacokinetic parameters (volume of distribution, bioavailability, and clearance) were obtained using a previously published population pharmacokinetic two-compartment model of dabigatran etexilate. 17 Simulated individual pharmacokinetic parameters for the first compartment [clearance (CL), central volume of distribution (V1) and bioavailability (F)] were determined for patients included in the study cohort using the previously published nonlinear mixed-effects model using Phoenix NLME Version 7. AUC _ss_ for a 24-hour period, the exposure parameter, was derived using these simulated estimates and the dosing data obtained from the PC. The second-compartment parameters were fixed (intercompartmental clearance [ *Q* = 35.5 L/h] and volume distribution of peripheral compartment [ *V*_per_ = 345 L]).

### Patient Outcomes

The outcomes of interest were 1 any admission to hospital for hemorrhage or 2 thromboembolism/CVA. These were extracted from the New Zealand Ministry of Health National Minimum Dataset (NMDS). 20 The NMDS is the national record of all public and private hospital discharge information, including coded clinical data for admissions longer than 4 hours for all of the New Zealand population. This database uses the International Statistical Classification of Diseases and Related Health Problems Tenth Revision, Australian Modification (ICD-10-AM) to record the diagnosis. 21 The date of admission to hospital was also extracted and aligned with the preceding dispensing of dabigatran etexilate for that patient. Patients were followed from their first dispensing of dabigatran etexilate until the date of hospitalization, cessation of dabigatran etexilate treatment, or study end.

### Statistical Analyses

Continuous variables were tested for normal distribution by the skewness and kurtosis test. Normally distributed data are presented as the mean ± standard deviation and nonnormally distributed data as the median (interquartile range, IQR). Categorical variables were expressed as percentages.

To evaluate the exposure–response relationship, patients were stratified into AUC _ss_ exposure quartiles (Q1–Q4). As there were differences in individual patient follow-up time, the risk of hemorrhage or thromboembolism/CVA between AUC _ss_ quartiles was compared using Poisson regression. 22 The resulting estimates were expressed using incidence rate ratios (IRRs; 95% confidence intervals [CIs]). 22 As exposure changed over time the standard hazard functions could not be fitted and rates were calculated per unit time exposure from which rate ratios (which are unitless) were calculated. Models were adjusted for known potential pharmacodynamic confounders: gender, age 75 to 79 years, age 80 years and over, Māori and Pacific peoples ethnicities, and deprivation rating (continuous). 23 Results were considered statistically significant if *p* < 0.05.

Statistical analyses were performed using Stata/IC (Version 14.2, StataCorpLP, TX, United States).

## Results

### Patient Characteristics

There were 2,660 individual patients identified in the databases who had been dispensed dabigatran etexilate with a diagnosis of atrial fibrillation (AF), aged 18 years or more, and who had one or more serum creatinine and bodyweight measurement recorded. The median age of patients in this cohort was 73 years (IQR: 66–79.5 years) and 1,525 (57.3%) were male. The median bodyweight was 85 kg (IQR: 72–100 kg). The median creatine clearance was 74.1 mL/min (IQR: 56.1–98.2). Overall patient covariate information is displayed in Table 1 .

**Table 1:** Individual patient covariate information

Covariate	*n*	%	Total exposure (person-years)
**Sex**			
Male	1,525	57.3	3,023
Female	1,135	42.7	2,156
**Age**			
< 65 y	554	20.8	952
65–74 y	922	34.7	1,836
75–79 y	519	19.5	1,043
> 80 y	665	25.0	1,318
**Ethnicity**			
European	2,098	78.9	4,075
Māori	315	11.8	587
Pacific peoples	65	2.4	129
Asian	24	0.9	63
MELAA	3	0.1	7
Other ethnicity	155	5.8	287
Heart failure	411	15.5	845
**Renal status**			
Severe impairment (CrCl < 30 mL/min)	43	1.6	58
Moderate impairment (30 ≤ CrCl < 50 mL/min)	425	16.0	732
Mild impairment (50 ≤ CrCl < 80 mL/min)	1,036	39.0	2,081
No impairment (80 ≤ CrCl < 120 mL/min)	818	30.8	1,631
No impairment (CrCl > 120 mL/min)	338	12.7	647
**Co-prescribed medications**			
Verapamil	42	1.6	85
Amiodarone	165	6.2	257
Proton pump inhibitor	1,186	44.6	2,268
**New Zealand deprivation score**			
1–Most deprived	154	5.8	299
2	165	6.2	311
3	224	8.4	471
4	206	7.7	372
5	183	6.9	346
6	212	8.0	397
7	224	8.4	430
8	192	7.2	372
9	232	8.7	458
10–Least deprived	200	7.5	378
Not recorded	668	25.1	1,315
**Formulation dispensed (at baseline)**			
75 mg	43	1.6	2
110 mg	1,375	51.7	127
150 mg	1,242	46.7	95
**Formulation dispensed (for all dispensings)**			
75 mg	1,189	1.8	80
110 mg	34,670	53.1	2,631
150 mg	29,374	45.0	2,438

Abbreviation: MELAA, Middle Eastern, Latin American or African ethnicity.

### Patient Outcomes

Approximately 4% of patients required hospitalization due to an adverse event occurring while being treated with dabigatran etexilate; there were 87 (3.3%) hemorrhagic events with a median age of 74 years (IQR: 70–81 years) and 29 (1.1%) thromboembolic/CVA events with a median age of 72 years (IQR: 68–79 years).

### Exposure-Derived Area Under the Curve

There were 65,233 individual prescriptions for dabigatran etexilate with a total 5,149 person-years supplied. The median AUC _ss_ was 2.0 mg h/L (IQR: 1.7–2.3 mg h/L) and when stratified by quartile each had the following results:

- Q1 had a mean of 1.48 mg h/L (range: 0.79–1.69 mg h/L),
- Q2 had a mean of 1.83 mg h/L (range: 1.70–1.96 mg h/L),
- Q3 had a mean of 2.11 mg h/L (range: 1.97–2.26 mg h/L),
- Q4 had a mean of 2.74 mg h/L (range: 2.27–12.76 mg h/L).

Simulated pharmacokinetic parameters are summarized in Table 2 . Those with an AUC _ss_ in the second and third quartiles had a reduced risk of hemorrhage [IRR: 0.51; 95% CI: 0.32–0.79; *p* = 0.003] and thromboembolism/CVA [IRR: 0.34; 95% CI: 0.16–0.76; *p* = 0.008] respectively, while those with an AUC _ss_ in the fourth quartile had an increased risk of hemorrhage [IRR: 1.68; 95% CI: 1.18–2.38; *p* = 0.004] ( Table 3 ).

**Table 2:** Summary of simulated pharmacokinetic parameters ( *n* = 65,233)

Simulated parameter	Mean (SD)	Median	IQR
CL (ml/min)	69.3 (17.6)	68.8	56.9–81.2
*V*_1_ (L)	710.1 (117.6)	697.4	630.0–775.1
F	1.0 (0.1)	1.0	0.9–1.0

Abbreviations: CL, clearance; F, bioavailability; IQR, interquartile range; SD, standard deviation; *V*_1_ , central volume of distribution.

Note: Fixed parameters; *Q* (35.5L/h) and *V*_per_ (345 L).

**Table 3:** Poisson regression expressed as adjusted incidence rate ratios (IIRs) and 95% confidence interval (95% CI) of hemorrhage and thromboembolism/CVA by AUC _ss_ quartiles ( *n* = 65,233)

	Hemorrhage			Thromboembolism/CVA		
AUC _ss_	IRR (95% CI)	*z*	*p* > | *z* |	IRR (95% CI)	*z*	*p* > | *z* |
Quartile 1	0.9 (0.61–1.33)	−0.52	0.606	1.31 (0.73–2.34)	0.89	0.371
Quartile 2	0.51 a (0.32–0.79)	−3.01	0.003	1.49 (0.87–2.55)	1.45	0.146
Quartile 3	1.13 (0.81–1.59)	0.71	0.478	0.34 a (0.16–0.76)	−2.66	0.008
Quartile 4	1.68 a (1.18–2.38)	2.89	0.004	1.20 (0.68–2.12)	0.62	0.536

Abbreviations: AUC _ss_ , area under the curve at steady state; CVA, cerebrovascular accident; IRR, adjusted incidence rate ratio for potential confounders: gender, age 75 years and over, Māori and Pacific peoples ethnicities and deprivation rating (continuous).

a Statistically significant.

## Discussion

This study demonstrates the feasibility of using measures of dabigatran etexilate pharmacokinetic exposure to optimize dosing and potentially improve patient outcomes.

The present study utilizes a novel approach of combining individual patient data, treatment outcomes, and a previously developed population pharmacokinetic model of dabigatran etexilate. AUC _ss_ data for each individual patient were derived and adjusted IRR at each AUC _ss_ quartile calculated. We observed that there was a greater risk for a hemorrhage observed for those patients with an AUC _ss_ in the fourth quartile, while there was a protective effect observed in the second quartile. In relation to thromboembolism, there was also a protective effect observed for those patients with an AUC _ss_ in the third quartile. Therefore, adjusting doses to achieve an AUC _ss_ that is in the range of 1.70 to 2.26 mg h/L may result in better outcomes for patients.

Additionally, the median age of our cohort was 73 years, which is older than that of patients enrolled in the dabigatran etexilate clinical trial. 24 It is acknowledged in the literature that elderly patients are at an overall increased risk of adverse outcomes with other studies having demonstrated that increasing age was a risk factor for having increased levels of dabigatran etexilate. 13 25 Therefore, the present study is able to provide a basis for developing reference ranges for optimal dosing for this at-risk and understudied population.

Currently it is not clear if TDM would be cost-effective in the clinical setting. It has been recently reported that with the rapidly growing use of DOACs there is increasing debate about the utility of point-of-care testing. 13 Despite these tests being available they are more expensive than traditional coagulation tests which may limit their utilization. With traditional coagulation tests having limited use in clinical practice, due to either poor sensitivity or standardization, 9 it has been suggested that point-of-care use in selected clinical situations may counterbalance their cost. 13 Additionally, to achieve the target AUC _ss_ , the dosing schedule may need altering (for example, extending or reducing dose intervals or combining different available formulation strengths) and dose simulations might clarify this point. Therefore, the present study can aid in the future economic evaluation for cost-effectiveness of TDM utility for dabigatran etexilate by providing treatment reference ranges conducive to favorable patient outcomes.

This study provides a preliminary clinical therapeutic reference range for dabigatran etexilate to help guide further studies investigating optimal dosing. Additionally, we are able to provide a real-world study population that has sufficient numbers to examine subtherapeutic treatment, a gap in knowledge identified in the literature. 12 With dabigatran etexilate TDM suggested to possibly reduce major bleeds by 30 to 40%, compared with well-controlled warfarin, 6 this study expands on this by providing a reference range that could be translated into the clinical setting along with a possible decrease in thrombotic events.

The limitations of this study include the NMDS only capturing patient data for those who require in-patient hospitalization for a duration of more than 4 hours. Therefore, any outcomes of interest that did not meet these criteria, for example, a hemorrhage or thromboembolism/CVA that resulted in death without an in-patient hospitalization, would not be included in the dataset resulting in possible underestimations. Additionally, there is the possibility of errors in the clinical information from the NMDS and primary-care dataset. These errors could result in inclusion or exclusion of clinical outcomes of interest. Although, it has been reported that there is high sensitivity when using the International Classification of Diseases, Ninth Revision, Clinical Modification (ICD-9-CM) to identify hemorrhagic events with 93% sensitivity and 88% specificity to identifying a definite major hemorrhagic event. 26 Similarly, it has been reported that using ICD-10 to identify thromboembolism/CVA has a positive predictive value (PPV) of close to or greater than 90% and therefore adequate to identify thromboembolism/CVA. 27 We assumed that the ICD-10-AM used to identify hemorrhagic events had high sensitivity and specificity and those used to identify thromboembolism/CVA had a high PPV. These limitations contribute to background variability but would not be expected to contribute to a systematic bias. Additionally, the PC only provides information related to the dispensing of medications and it is not possible to confirm if the patients within this cohort have adhered to the prescribed regimen. Also, the population pharmacokinetic model utilized did not include covariate effects for other drug interactions (such as atorvastatin, rifampicin, clarithromycin) as there were no published validated models. Although risk factors were included in the exposure model utilized, HAS-BLED and CHA2DS2-VASc covariates were not. However, known risk factors in our population were included (such as ethnicity, increasing age, and deprivation score). The main strength of this study is the inclusion of a large cohort of patients with sufficient sample size to provide adequate information about dabigatran etexilate exposure response.

## Conclusion

This retrospective cohort demonstrated that there is a relationship between dabigatran etexilate exposure and adverse response in real-world patients. It has established that it is feasible to provide guidance for optimal dosing to improve outcomes for patients with AF. This is particularly relevant for elderly patients who are at greater risk of adverse treatment outcomes.
